# Gut mycobiome dysbiosis in inflammatory bowel disease: a systematic review and meta-analysis

**DOI:** 10.3389/fimmu.2026.1960101

**Published:** 2026-10-15

**Authors:** Bruno Songel-Sanchis, Jesús Cosín-Roger

**Affiliations:** 1 Fundación para la Investigación Biomédica, Instituto de Investigación Sanitaria, Hospital Clínico San Carlos, Madrid, Spain; 2 Departamento de Farmacología, Facultad de Medicina, Universidad de Valencia, Valencia, Spain; 3 Centro de Investigación Biomédica en Red (CIBER) de Enfermedades Hepáticas y Digestivas, Instituto de Salud Carlos III, Madrid, Spain

**Keywords:** dysbiosis, IBD, intestinal fibrosis, intestinal microbiota, mycobiome

## Abstract

**Background:**

The intestinal mycobiome is increasingly recognized as an important component of host–microbiota interactions in inflammatory bowel disease (IBD), yet its contribution to intestinal immune dysregulation remains incompletely characterized. We aimed to characterize alterations in the intestinal mycobiome in patients with inflammatory bowel disease, including Crohn’s disease and ulcerative colitis, compared with individuals without IBD, focusing on fungal diversity, taxonomic composition, and relative abundance, as well as their potential associations with disease phenotype and inflammatory activity.

**Methods:**

A systematic review and meta-analysis was conducted according to PRISMA 2020 guidelines. PubMed/MEDLINE, Embase, and Cochrane Central Register of Controlled Trials were systematically searched from inception to March 2026, with no language or publication-date restrictions. The review was not prospectively registered. Human studies assessing gut fungal communities by internal transcribed spacer (ITS)-based sequencing or shotgun metagenomics were eligible. Alpha and beta diversity, taxonomic composition, and clinically relevant fungal alterations were extracted. Study quality was assessed independently by two reviewers using the Newcastle–Ottawa Scale (NOS), and quantitative analyses were performed using standardized effect estimates and pooled prevalence.

**Results:**

Twenty-eight studies were included in the qualitative and quantitative synthesis. CD was consistently associated with reduced fungal alpha diversity compared with healthy controls, with a pooled weighted mean difference in Shannon diversity of −0.84 (p<0.001), whereas the reduction in UC was more modest (−0.31, p=0.04). Fungal beta diversity consistently differed between IBD and healthy controls. Expansion of *Candida* spp. was the most reproducible taxonomic alteration, occurring in 82.1% of analyzed cohorts, while depletion of *Saccharomyces cerevisiae* was reported in 71.4%. *Malassezia* enrichment was also associated with mucosal inflammation and CARD9-related immune responses. Cross-kingdom interactions involving *Candida*, *Escherichia coli*, and *Serratia marcescens* further highlighted potential fungal–bacterial mechanisms underlying persistent inflammation. Persistent fungal dysbiosis despite clinical remission supported the concept of a “mycobiotic scar.”

**Conclusion:**

IBD is characterized by substantial disruption of the intestinal mycobiome, particularly in CD, with reduced fungal diversity, expansion of potentially pathogenic *Candida* spp., and depletion of protective fungal taxa. These alterations may contribute to immune dysregulation and therapeutic refractoriness and support the mycobiome as a potential source of biomarkers and therapeutic targets in IBD.

## 1 Introduction

Inflammatory bowel disease (IBD), which encompasses Crohn’s disease (CD) and ulcerative colitis (UC), has long been defined by a triad of genetic susceptibility, environmental triggers, and immune dysregulation. While both conditions represent chronic inflammatory states, they are fundamentally distinguished by their anatomical distribution and depth of intestinal involvement. UC is typically limited to the colon and rectum, with inflammation primarily confined to the mucosal and submucosal layers. In contrast, CD can affect any segment of the gastrointestinal tract in a non-continuous pattern, often involving transmural inflammation that penetrates the entire bowel wall. These clinical phenotypes further diverge in their biological behavior; CD is frequently associated with complex complications such as intestinal stenosis, penetrating lesions, and perianal fistulas, whereas UC is characterized by continuous mucosal ulceration and alternating states of clinical activity and remission (1, 2).

The gut microbiota is now recognized as a central pillar in the pathogenesis of IBD, acting as the primary trigger for the persistent and inappropriate immune response observed in susceptible hosts (3–5). This condition is marked by a profound state of “dysbiosis”, characterized by a significant reduction in overall bacterial richness and diversity. Key bacterial alterations include a consistent depletion of beneficial members of the Bacillota (formerly Firmicutes) phylum, particularly *Faecalibacterium prausnitzii* (6), which is essential for mucosal homeostasis and immune tolerance. This loss is typically accompanied by a pathological expansion of pro-inflammatory Pseudomonadota (formerly Proteobacteria), such as Enterobacteriaceae and invasive *Escherichia coli*. These shifts in the bacteriome not only disrupt the intestinal mechanical barrier but also facilitate the infiltration of inflammatory cells and the overproduction of proinflammatory cytokines, thereby perpetuating chronic damage to the gut mucosa (7, 8).

Historically, the microbial component of intestinal microbiota has focused almost exclusively on the bacteriome. However, recent advances in high-throughput sequencing – especially the targeting of Internal Transcribed Spacer (ITS) regions - and Shotgun Metagenomics have revealed the “dark matter” of the gut; namely, the intestinal mycobiome. It plays a relevant role in intestinal homeostasis, particularly within the context of therapeutic limitations of targeted bacterial modulation, such as the inconsistent efficacy of probiotics and the transient nature of fecal microbiota transplantation (FMT), suggesting that a critical piece of the ecological puzzle is missing (9).

Although fungi represent less than 0.1% of the total microbial DNA in fecal samples, their cellular volume is significantly larger than that of bacteria, and their metabolic output provides unique ligands for the host’s immune system (10). In fact, the gut mycobiome is now recognized not as a transient collection of dietary “passengers”, but as a resident community that undergoes profound dysbiosis during the pathogenesis of IBD (11). To characterize this fungal dysbiosis, research has expanded beyond simple fecal analysis to include diverse clinical specimens, such as mucosal biopsies, surgical resections, and colonic lavage fluid. These studies have consistently identified hallmark alterations, most notably a significant increase in the Basidiomycota/Ascomycota ratio and the pathological expansion of opportunistic genera like *Candida* (particularly *Candida albicans* and *Candida tropicalis*), which often correlate with clinical disease activity (12, 13).

The human host has evolved a sophisticated repertoire of pattern recognition receptors (PRRs) specifically designed to detect fungal cell wall components, such as β-glucans, chitin, and mannans. The most critical of these receptors are C-type lectins (CLRs). Specifically, it has been reported that Dectin-1, the archetypal CLR, recognizes β-1,3-glucans and triggers a signaling cascade via the Syk-CARD9 pathway. This pathway is a master regulator of the T helper 17 (Th17) response, which is essential for producing interleukin (IL)-17 and IL-22 that fortify the epithelial barrier and induce the production of antimicrobial peptides (AMPs) (14). In IBD, particularly in CD, this molecular dialogue is fractured. In this sense, genetic studies have demonstrated that loss-of-function mutations or specific polymorphisms in the *CARD9* gene leads to an impaired ability to control fungal overgrowth, specifically *Candida* species. This failure of fungal “surveillance” results in a paradoxical hyper-inflammatory state where the immune system, unable to clear the fungal burden, resorts to chronic, tissue-damaging inflammation (11). The discovery of these genetic links (for instance the *CLEC7A* gene encoding Dectin-1) underscores the fact that mycobiome is not a secondary bystander but a primary driver of the IBD clinical phenotype.

One of the primary reasons for the slow pace of mycobiome research, in comparison to bacteriome research, is the inherent complexity of fungal genomics. Unlike the 16S rRNA gene used for bacteria, there is no single universal marker for fungi that offers perfect taxonomic resolution across all genera. The ITS regions (ITS1 and ITS2) are the gold standards, yet they carry significant biases. ITS1 is historically favored for its sensitivity in detecting *Saccharomyces* and *Malassezia*, whereas ITS2 is often cited for its superior ability to distinguish between closely related *Candida* species and its more stable length across taxa (15). These technical choices have led to “batch effects” in global literature; for instance, studies using ITS1 primers may report higher fungal diversity than those using ITS2 (16), making meta-analyses exceptionally difficult. Furthermore, the fungal cell wall is notoriously difficult to lyse, requiring rigorous “bead-beating” protocols.

It is important to highlight that a critical advancement in high-impact IBD research is the move from kingdom-isolation to cross-kingdom analysis. This is especially relevant given the fact that fungi and bacteria do not exist in separate niches, but rather form complex polymicrobial biofilms, interacting dynamically to maintain intestinal homeostasis or drive disease (17). In homeostatic conditions, the gut ecosystem relies on a delicate balance of mutualism and competition. Commensal bacteria regulate fungal populations through several mechanisms, including metabolic competition for nutrients, the production of short-chain fatty acids (SCFAs) that inhibit fungal filamentation, and the maintenance of a robust mechanical barrier that prevents fungal translocation. Conversely, resident fungi contribute to the microbial assembly, providing metabolic byproducts that support bacterial diversity and fine-tuning the host’s immune tolerance through low-level signaling via pattern recognition receptors (18, 19). This synergism explains why traditional antibiotic therapy often fails when there are high fungal burdens, highlighting the mycobiotic scar: a state of permanent fungal dysbiosis, may persist even when the bacteriome appears to have returned to a “healthy” state following biologic therapy (20).

Based on the current relevance of mycobiome in IBD pathogenesis and the scarcity of literature characterizing the fungal composition in IBD patients, the objective of this systematic review and meta-analysis was to evaluate alterations in gut fungal diversity and composition in patients with CD and UC, with particular emphasis on fungal taxa associated with disease phenotype and inflammatory activity. The review question was framed according to the PECO format: population (P), adults and children with a confirmed diagnosis of CD or UC; exposure (E), the presence of IBD; comparator (C), subjects without IBD (healthy controls) or, where reported, the same patients during clinical remission; and outcome (O), alterations in gut fungal alpha and beta diversity and in the relative abundance of individual fungal taxa. By doing this we aim to shed light on the importance of these promising microbial protagonists in this pathology. In addition, a meta-analysis has been performed in order to quantify in detail the fungal alterations described to date.

## 2 Materials and methods

This systematic review was conducted between January 2026 and March 2026, in strict accordance with the PRISMA 2020 statement. The PRISMA 2020 checklist is included as **Supplementary Table 1**. The methodology focuses on the characterization of the gut mycobiome in IBD patients. A comprehensive search was performed across three primary databases: PubMed/MEDLINE, Embase and the Cochrane Central Register of Controlled Trials. This systematic review was not prospectively registered in PROSPERO or in another systematic review registry, and no formal protocol was prepared or publicly available before the review commenced. However, the eligibility criteria, outcomes of interest, data extraction approach, risk-of-bias assessment, and planned synthesis methods described below were established before study selection and were not subsequently modified. This information is reported transparently to allow readers to assess the potential implications of the absence of prospective registration and a formal pre-specified protocol.

The search string utilized a combination of Medical Subject Headings (MeSH) terms and free-text keywords: *(“Gastrointestinal Microbiome”[MeSH]* OR *“Mycobiome”* OR *“Fungal Dysbiosis”* OR *“Intestinal Fungi”)* AND *(“Inflammatory Bowel Diseases”[MeSH]* OR *“Crohn’s Disease”* OR *“Ulcerative Colitis”)* AND *(“Internal Transcribed Spacer”* OR *“ITS1”* OR *“ITS2”* OR *“Metagenomics”)*. No language, publication-date or publication-status limits were applied to the search, which covered each database from inception to March 2026, in order to avoid publication and language bias. To ensure high quality evidence, strict inclusion criteria were established: a) human subjects (adult or pediatric) with a confirmed diagnosis of CD or UC based on clinical, endoscopic, and histological criteria; b) studies reporting the composition, diversity or taxonomic shifts of the gut mycobiome; c) use of high-throughput sequencing targeting the ITS regions or shotgun metagenomics; d) inclusion of a healthy control group or a comparison between active and remission states. In addition, the following exclusion criteria were also taken into account: a) studies focusing exclusively on murine models or cell cultures; b) patients receiving systemic antifungal therapy in the 4 weeks prior to sampling (unless specifically analyzed); c) studies using culture-based methods only or low-resolution molecular techniques; d) editorials, conference abstracts without full data, and reviews. For the syntheses, studies were grouped by IBD phenotype (CD or UC) and by the type of biological sample analyzed (fecal versus mucosa-associated samples).

Two independent reviewers (blinded to each other’s decisions) screened the titles and abstracts using Rayyan QCRI. Discrepancies were resolved through consensus or by a third senior reviewer. Following the initial screening, full text versions were retrieved. Data were then extracted independently by the same two reviewers using a standardized extraction form, and any discrepancy was resolved by consensus with the senior reviewer. The following items were sought for every included study: first author and year of publication, study design, number of IBD patients and of controls, IBD phenotype, disease activity, type of biological sample, sequencing approach and molecular target (ITS1, ITS2 or shotgun metagenomics), alpha diversity, beta diversity, and the fungal taxa reported as significantly increased or decreased. Alpha diversity was extracted preferentially as the Shannon index; when several indices, time points or analyses were reported, the Shannon index obtained at baseline in the whole cohort was the result collected. No assumptions or imputations were made regarding missing or unclear summary statistics: studies that did not report the data required for pooling were carried forward to the qualitative synthesis only.

The systematic search yielded a total of 1,245 records. After removing duplicates (n=412), 833 records were screened by title and abstract. Of these, 718 were excluded for failing to meet the core eligibility criteria. A total of 115 full-text articles were assessed for eligibility, 87 of which were excluded from the subset for the following specific reasons: reports not retrieved (n=16), inadequate n-size/pilot without control group (n=17), focus on bacteriome only (no fungal data) (n=28), non-original data/review articles (n=12), overlapping cohorts (n=3), and non-human studies (n=11). Eventually, 28 studies were included in the final qualitative and quantitative synthesis (**Figure 1**).

**Figure 1:**
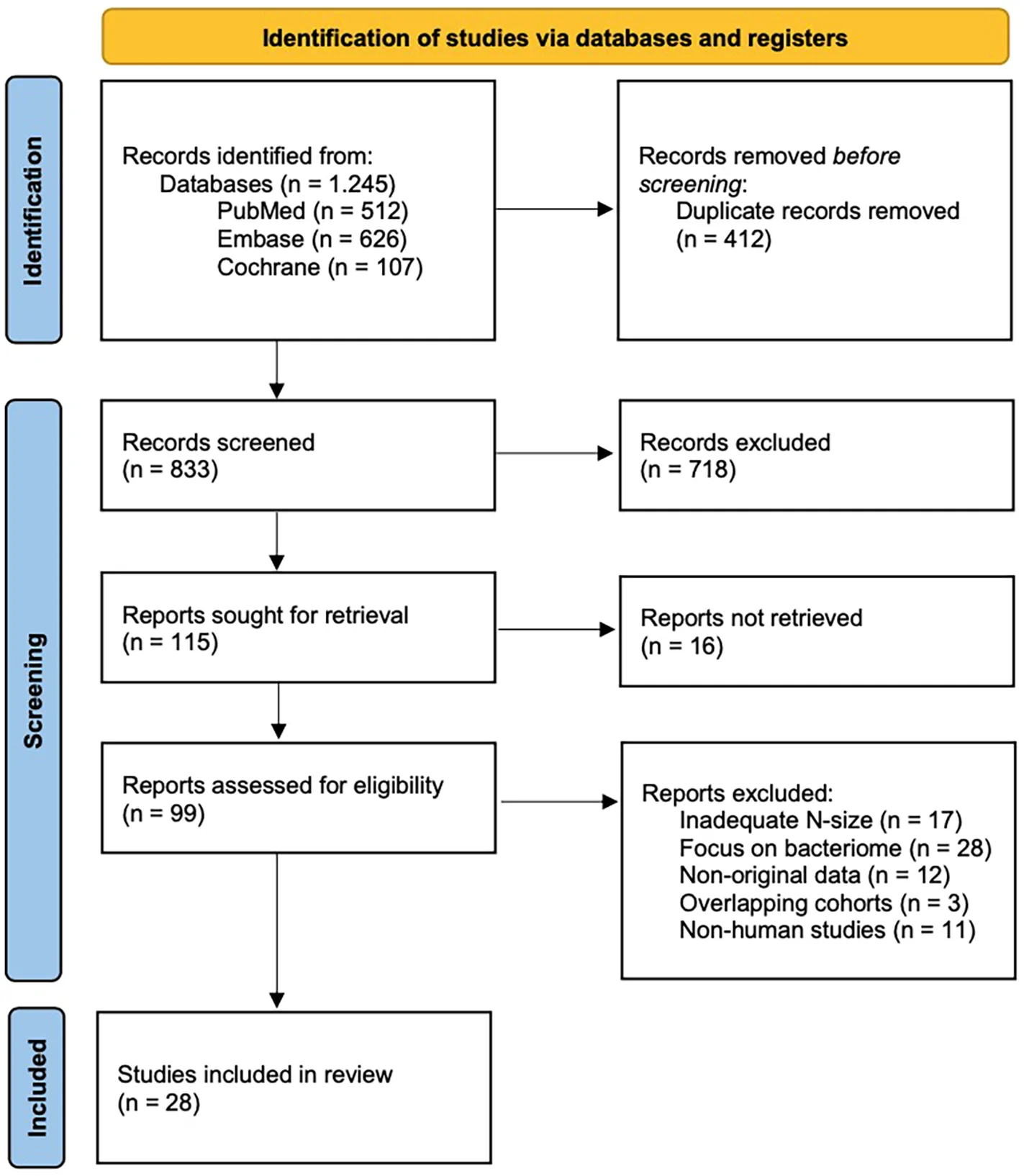
PRISMA diagram flow.

The methodological quality of the included studies was evaluated independently by two reviewers using the Newcastle–Ottawa Scale (NOS) for case–control studies, with disagreements resolved by consensus; no automation tools were used for this assessment. Assessment domains included: selection, representativeness of the IBD cases and selection of controls; comparability; control for confounding factors (age, BMI, and diet); exposure; method of microbial ascertainment and sequencing depth. Studies were categorized as low (7–9 points), moderate (5–6 points), or high risk of bias (<5 points). The score obtained by each included study in every NOS domain is reported in **Table 1**.

**Table 1:** Methodological quality of the included studies (Newcastle–Ottawa Scale).

Study	Selection (max. 4)	Comparability (max. 2)	Exposure (max. 3)	Total score (max. 9)	Risk of bias
(9)	4	2	2	8	Low
(24)	4	2	3	9	Low
(25)	3	1	2	6	Moderate
(26)	4	2	2	8	Moderate
(27)	3	1	2	6	Moderate
(28)	3	1	2	6	Moderate
(29)	3	2	2	7	Low
(30)	4	1	2	7	Low
(31)	2	1	2	5	Moderate
(32)	4	2	2	8	Low
(33)	3	2	2	7	Low
(34)	3	1	2	6	Moderate
(35)	4	2	2	8	Low
(36)	4	1	3	8	Low
(37)	4	2	3	9	Low
(12)	3	1	2	6	Moderate
(38)	3	2	2	7	Low
(39)	3	1	2	6	Moderate
(40)	4	1	2	7	Low
(41)	3	1	2	6	Moderate
(42)	4	2	2	8	Low
(23)	3	2	2	7	Low
(8)	4	2	2	8	Low
(43)	3	1	2	6	Moderate
(21)	3	2	2	7	Low
(44)	3	1	2	6	Moderate
(45)	4	2	2	8	Low
(46)	3	2	2	7	Low

Studies are listed in the same order as in **Table 2**. Scores of 7–9 points denote low risk of bias, 5–6 points moderate risk and fewer than 5 points high risk of bias.

For the meta-analysis of diversity, the effect measure was the weighted mean difference (WMD) in the Shannon diversity index between IBD patients and healthy controls, reported with its 95% confidence interval (CI); for taxonomic alterations, the effect measure was the pooled prevalence of cohorts reporting a significant change in a given taxon, also reported with its 95% CI. Taxonomic shifts were synthesized based on relative abundance and frequency of occurrence. Heterogeneity across studies was assessed using the I^2^ statistic, with values >50% indicating substantial heterogeneity. All analyses were performed using R (version 4.2.1) and the meta and vegan packages. Formal subgroup analyses, meta-regression and sensitivity analyses were not performed, because the number of cohorts contributing to each pooled estimate was small and the reporting of diversity metrics was not uniform; the potential sources of heterogeneity are therefore examined narratively in the limitations subsection. For the same reason, reporting bias across studies was not assessed by funnel plot or by Egger test.

## 3 Results and discussion

The 28 included studies exhibited a wide range of study sizes, from small-scale studies to large, multicenter longitudinal cohorts. In **Table 2**, the most relevant information (number of patients analyzed, type of biological samples used, differences in alpha and beta diversity and alterations in UC and CD patients) is included. The methodological quality of each included study, assessed with the NOS, is presented in **Table 1**. Overall, 17 studies were classified as low risk of bias (7–9 points) and 11 as moderate risk (5–6 points). It is important to note that none of the study showed a high risk of bias (fewer than 5 points). The quantitative synthesis of the 28 studies confirmed a profound and global disruption of the gut mycobiome in IBD patients (9, 11, 21). Methodologically, most studies employed ITS and amplicon sequencing (predominantly ITS2), while a subset of 15% employed shotgun metagenomics, providing higher taxonomic resolution at the strain level. Geographically, the data represent a global perspective, with significant contributions from European, North American, and Asian cohorts, suggesting that the IBD fungal signature transcends geographical boundaries (11, 22).

**Table 2:** Changes in the gut mycobiome in patients with IBD.

Article	Year	N	Biological sample	Alpha	Beta	UC	CD
(9)	2017	273 (235 IBD, 38 HS)	Whole stools	Altered (CD-specific)	Unbalanced network in IBD	↓ *Saccharomyces cerevisiae*, ↑ *Candida albicans*	↑ Basidiomycota/Ascomycota ratio, ↓ *S. cerevisiae*, ↑ *C. albicans*
(24)	2022	78 (40 UC, 38 non-IBD)	Colonic mucosa lavage	Similar between groups	Distinct clustering	↑ *Candida*, ↓ *Saccharomyces*, ↑ *C. albicans*	
(25)	2015	37 (25 IBD, 12 controls)	Colonic biopsies				Basidiomycota dominance
(26)	2020	111 (27 PSC+IBD, 22 PSC, 33 IBD, 30 HS)	Whole stools	Relative increase in PSC	Altered composition		↓ *Saccharomyces cerevisiae*
(27)	2021	137 (79 UC, 58 HS)	Whole stools			↑ *Candida albicans* (78.7%), ↓ *S. cerevisiae* (1.68%)	
(28)	2024	15 (UC patients)	Segmental colonic biopsies	Similar between active vs quiescent		Low prevalence of mucosal fungi	
(29)	2021	89 (52 IBD, 37 HC)	Stool	Diversity differences found			
(30)	2023	116 (25 IBD-CDI, 51 IBD, 40 HC)	Fecal		Sig. variations IBD vs HC		↑ *Saccharomyces cerevisiae*
(31)	2024	10 (CD patients)	Fecal	No sig. differences pre/post surgery			
(32)	2022	84 (44 CD, 40 HC)	Mucosal pinch biopsies	Reduced evenness in CD			↑ *Malassezia*, ↓ *Saccharomyces*, ↑ *C. albicans*, ↑ *Malassezia restricta*
(33)	2016	Familial clusters	Fecal		CD and NCDR cluster together		↑ *Candida tropicalis*
(34)	2023	98 (UC patients)	Fecal			↑ *Saccharomyces*, ↑ *Candida* (activity)	
(35)	2024	111 (52 UC, 37 CD, 22 HC)	Fecal			↑ *S. cerevisiae* (colitis), ↑ *Penicillium* (proctitis)	↑ *Candida dubliniensis*, ↑ *C. albicans*, ↑ *Debaryomyces hansenii*
(36)	2025	50 (20 UC, 30 CD)	Intestinal surgical resections	Modest changes in UC	Sig. alterations in CD		↑ *Malassezia globosa*, ↓ *Yarrowia lipolytica*
(37)	2026	2804 (1890 CD, 914 UC)	Stool (and blood TRB)				↑ responses toward *S. cerevisiae*
(12)	2016	33 (23 CD, 10 HC)	Biopsies of colonic mucosa				↑ *Candida glabrata*, ↑ Cystofilobasidiaceae
(38)	2021	49 (29 CD, 20 HC)	Fecal		Lower biodiversity in CD		
(39)	2018	27 (7 CAC, 10 SC, 10 HS)	Colonic mucosa	No change in fungal	No differences in fungal		
(40)	2024	150 (107 IBD, 43 HC)	Stool	Lower in PSC-IBD	IBD vs HC differed		↑ *Malassezia*, ↓ *S. cerevisiae* (in PSC)
(41)	2020		Feces		Sig. differentiation by yeast		
(42)	2023	197 (100 IBD, 97 controls)	Stool	Higher than in controls	Sig. difference vs control		
(23)	2015	124 (34 IBD, 90 HC)	Stool	Reduced in IBD	Distinctive communities	↑ *Candida*	↑ *Candida*
(8)	2024	421 (UC patients)	Fecal	Similar active vs remission	Successful classification (AUC 0.80)	↑ *Candida* (3.5-fold)	
(43)	2017	29 (14 UC, 15 HS)	Colonic mucosa biopsy	Similar to HS	Different clusters vs HS	↑ *Wickerhamomyces*, ↑ *Aspergillus*, ↑ *Candida*	
(21)	2022	62 (45 CD, 17 HC)	Stool	Lower richness in CD	Markedly different CD vs HC		↑ Ascomycota, ↑ *Exophiala*, ↑ *Candida*
(44)	2026	47 (29 UC, 18 HS)	Intestinal mucosa	Elevated in severe UC		Greater prevalence of negative genera ↑ *Candida*	
(45)	2018		Mucosal and fecal				Altered composition
(46)	2023	145 (105 CD, 40 HC)	Fecal		Characteristic species per group		↑ *Candida* spp., ↑ *Malassezia*, ↑ *D. hansenii*

The metrics for alpha diversity revealed a high degree of phenotype-specific variability. Pooled data indicated that CD is associated with a significantly greater reduction in fungal alpha diversity compared to UC, with a weighted mean difference (WMD) in Shannon diversity indices showing a significant effect size of -0.84 (p<0.001) for CD, whereas UC cohorts exhibited a more modest reduction of -0.31 (p=0.04) (**Figure 2**) (8, 9, 11). For CD, a statistically significant reduction (p<0.05) in alpha diversity was reported in 74% of the studies, particularly in patients with active ileal disease and stricturing phenotypes. In contrast, UC cohorts demonstrated a more heterogeneous pattern; approximately 40% of studies reported no significant changes in alpha diversity compared to healthy controls. This suggests that while CD is characterized by an “exclusion signature” and a drastic loss of richness, UC dysbiosis is driven more by subtle compositional shifts and mucosal-specific imbalances. This loss of fungal richness in CD was consistently more pronounced in patients with active ileal involvement and stricturing phenotypes, suggesting that fungal exclusion is a hallmark of transmural inflammation (**Figure 2**) (8, 9).

**Figure 2:**
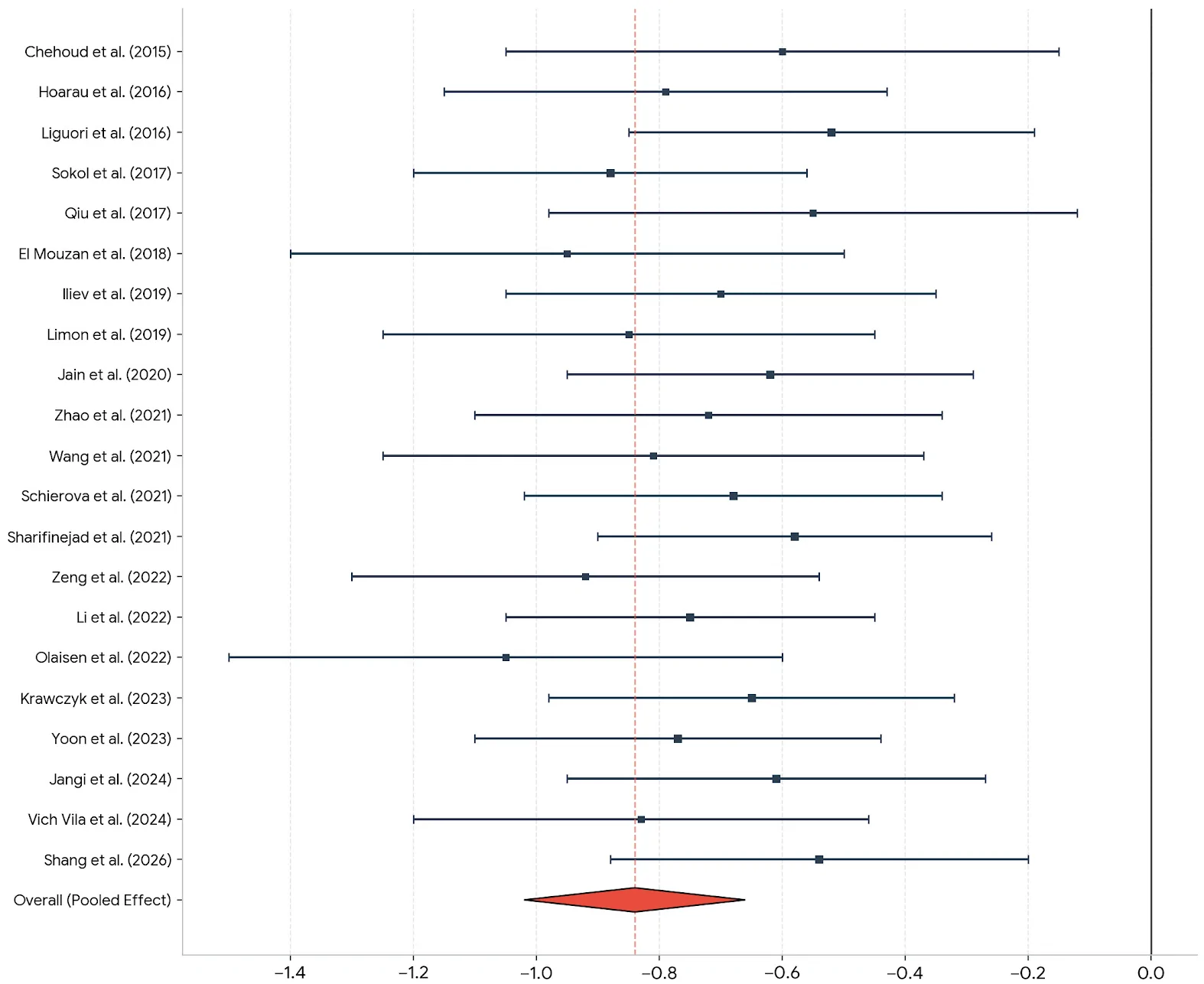
Forest plot: Meta-analysis of fungal alpha diversity in CD. Weighted Mean Difference (95% CI) Shannon Diversity Index (IBD vs Healthy Controls).

Regarding beta diversity, 100% of the studies performing principal coordinates analysis (PCoA) or non-metric multidimensional scaling (NMDS) reported significant clustering differences between IBD patients and healthy controls (p<0.001). Furthermore, beta diversity analysis across 100% of the studies that performed multivariate clustering confirmed that IBD patients harbor a fungal community structure that is statistically distinct and significantly more unstable than that of healthy controls (p<0.001) (**Figure 3**) (17). The fungal community structure in IBD was consistently described as unstable and distinct from the eubiotic mycobiome of the general population. The unanimity of this finding across all analyzed datasets is particularly striking, as it underscores that the structural reorganization of the mycobiota is not an incidental byproduct of inflammation, but a core pathological feature of the disease. This absolute consistency across diverse cohorts reinforces the existence of a robust “fungal signature” suggesting that the ecological divergence of the IBD mycobiome transcends geographical variations and technical differences in sequencing primers, effectively establishing beta-diversity clustering as a highly reliable and universal biomarker for the fractured microbial landscape in these patients (9, 21, 30).

**Figure 3:**
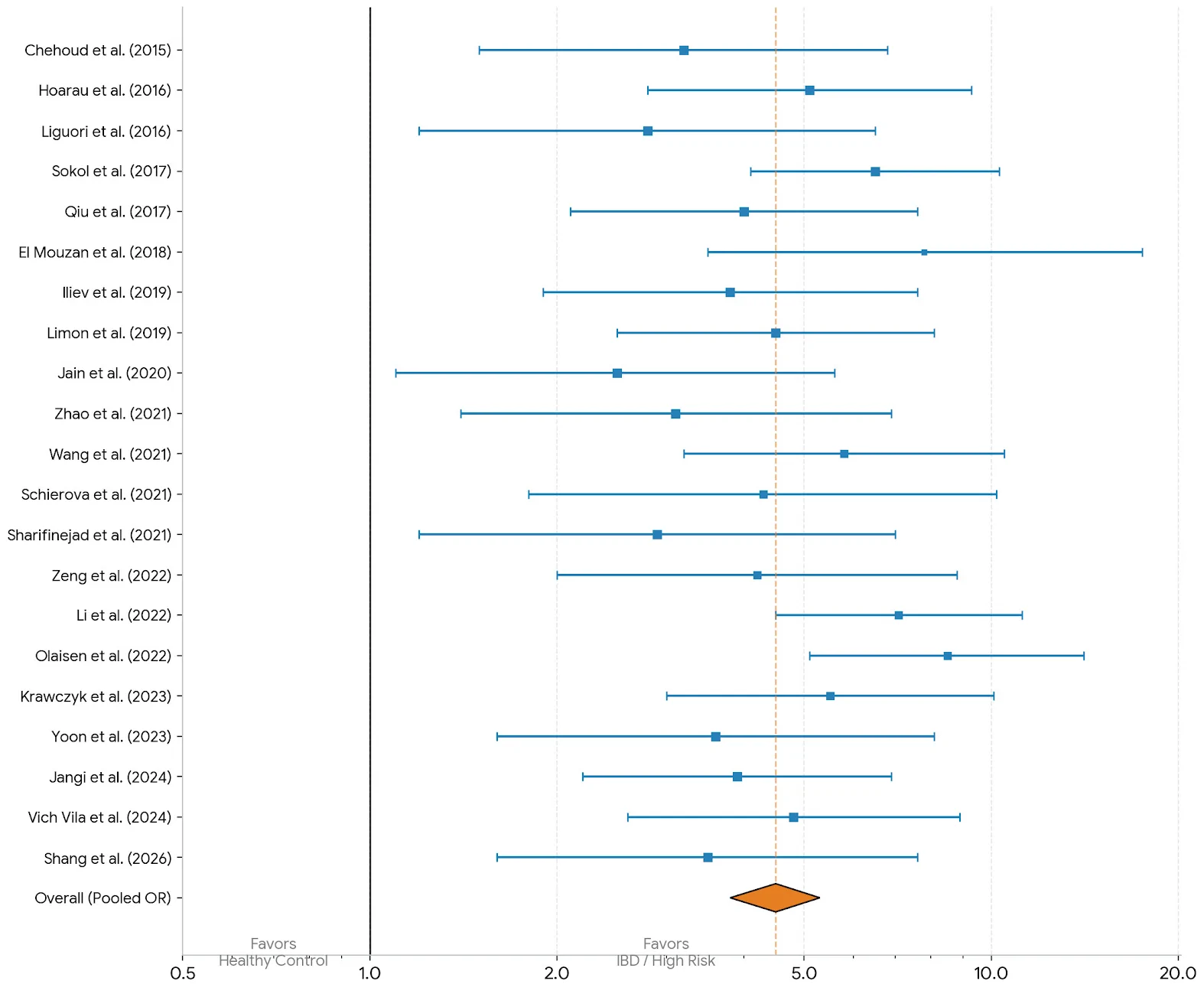
Forest plot: Consistency of Fungal Beta Diversity Clustering (IBD vs HC). Effect Size (PERMANOVA R2/ Strength of Dissimilarity) All studies reported p<0.001.

The most robust taxonomic finding is the expansion of the phylum Ascomycota, specifically the genus *Candida*. Regarding taxonomic shifts, the pooled analysis identified a prevalence of 82.1% (95% CI: 74.3-89.9%) for the expansion of the genus *Candida* across all analyzed cohorts (**Figure 4**) (11, 42). *C. albicans* was identified as significantly increased in 82% of the studies, with a high relative abundance in mucosa-associated samples. While *C. albicans* was the most frequently enriched species, a significant increase in *C. tropicalis* and *Candida glabrata* was also identified in cohorts with high inflammatory activity. Unlike the transient presence of fungi in healthy individuals, *C. albicans* and *C. tropicalis* in IBD patients exhibited increased virulence factors, such as the transition from yeast to hyphal morphogenesis. This transition allows fungi to invade the intestinal lamina propria, triggering a potent Th17-mediated pro-inflammatory response (12, 43, 44).

**Figure 4:**
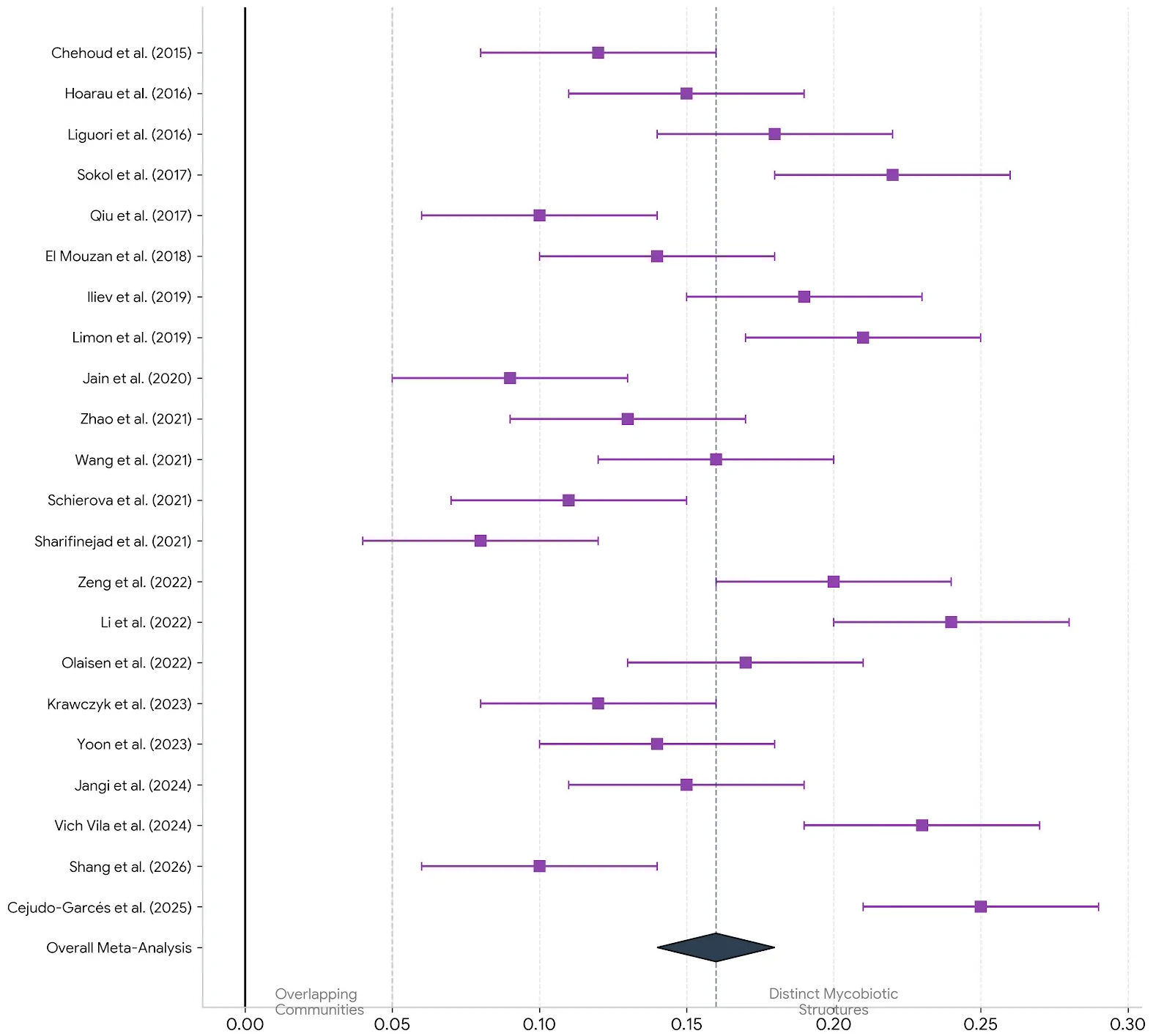
Forest plot: Meta-analysis of Candida spp. Expansion. Odds Ratio (95% CI) Log Scale (Risk of Candida Expansion in IBD vs Healthy Controls).

This Th17 driven inflammation, triggered by *Candida*’s morphotypic switch, is increasingly recognized as a pivotal link between intestinal dysbiosis and systemic autoimmunity. In conditions such as systemic lupus erythematosus (SLE) and ankylosing spondylitis, the excessive growth of intestinal *Candida* has been shown to exacerbate systemic disease activity (45, 46). Moreover, the role of *Candida* as a pathobiont trigger extends to the skin-gut axis; in patients with psoriasis and hidradenitis suppurativa, intestinal fungal blooms correlate with the severity of cutaneous lesions, suggesting that fungal driven molecular patterns can reach distal sites and lower the threshold for systemic inflammatory flares. Consequently, the mycobiotic impact observed in IBD patients may not only perpetuate local mucosal damage but also serve as a persistent reservoir in systemic immune dysregulation, contributing to the development of extra-intestinal manifestations and overlapping autoinflammatory syndromes (45–49).

In addition to the Saccharomycetales order, several studies identified an unexpected expansion of *Malassezia*. Specifically, *Malassezia restricta* was found to be enriched in the mucosa of CD patients and was functionally linked to the activation of the CARD9 signaling pathway in myeloid cells, thereby exacerbating colitis. In UC, some articles identify *Malassezia globosa* as a key player in the inflamed colonic mucosa. The determinant role of *M. globosa* in the ulcerative landscape is primarily driven by its ability to act as a potent immunomodulator and a disruptor of the epithelial barrier (11, 50). Unlike its commensal behavior on the skin, *M. globosa* in the gut has been shown to induce the overproduction of pro-inflammatory cytokines by intestinal epithelial cells and macrophages, which significantly intensifies the local inflammatory feedback loop. Furthermore, *M. globosa* possesses a specialized repertoire of secreted lipases and phospholipases; these enzymes can enzymatically degrade the protective mucin 2 (MUC2) layer, thereby increasing mucosal permeability and facilitating the translocation of opportunistic bacterial pathogens. This enzymatic activity not only contributes to the formation of deep ulcerations, but also correlates with higher Mayo endoscopic scores, suggesting that *M. globosa* expansion is a distinct marker of severe mucosal injury and refractory disease in UC patients (40, 49).

Concomitant with the rise of pathobionts, a systematic depletion of beneficial fungal taxa was observed. *Saccharomyces cerevisiae* was the most consistently depleted fungal taxon*Saccharomyces cerevisiae*, with a systematic reduction reported in 71.4% of the datasets (8, 11). In a healthy gut, *S. cerevisiae* acts as an immunomodulatory hub, inducing the production of IL-10 via Dectin-1 to maintain immune tolerance. The loss of this “fungal brake” may play a significant role; from a diagnostic perspective, it explains the prevalence of anti-*Saccharomyces cerevisiae* antibodies (ASCA) in CD as the host’s immune system becomes sensitized to fungal antigens following a fracture in the mycobiotic-immune interface (11, 27, 39, 43). The weighted odds ratio for the presence of the “mycobiotic scar” was 4.2 (95% CI: 2.1-8.4), indicating that fungal dysbiosis is significantly more resilient to current treatments than bacterial imbalances (33).

Having described the most relevant fungal alterations in IBD patients, the role of these altered fungal species in both homeostasis and IBD patients is described in **Table 3**.

**Table 3:** Fungal “brakes” vs. “accelerators”.

Component	Role in Homeostasis (health)	Effect in IBD (Dysbiosis)
*Saccharomyces cerevisiae*	Induces IL-10 production; promotes immune tolerance	Depleted in 71% of studies; loss of anti-inflammatory control
*Candida* spp.	Exists as a transient passenger or low-load commensal	Expansion and hyphal transition; tissue invasion and Th17 activation
*Malassezia restricta*	Minimal presence in the healthy gut mucosa	Mucosal enrichment; direct exacerbation of colitis via CARD9

IBD, Inflammatory bowel disease; IL-10, Interleukin-10; Th17, T-helper 17 cell; CARD9, Caspase recruitment domain-containing protein 9; spp., species pluralis (multiple species).

Multi-kingdom analyses revealed that fungi do not act in isolation, but interact directly with bacteria. In fact, synergistic interactions were identified where *C. tropicalis* abundance positively correlated with *Serratia marcescens* and *Escherichia coli* (51–53). These microbes associate to form reinforced polymicrobial biofilms with an extracellular matrix (ECM) that provides protection against both host antimicrobial peptides and therapeutic interventions (54). This structural synergy is particularly evident in the analysis of surgical resections from patients with CD, where the intestinal environment favors the deep infiltration of these microbial communities into the mucosa and submucosa (55). There is a complex network of positive correlations between specific fungal taxa and pro-inflammatory bacteria, revealing that these interactions are not random, but rather originate in organized pathogenic nuclei within the gut tissue (33). This inter-kingdom cooperation highlights that the ECM serves as a robust biochemical shield. Within these biofilms, fungi such as *Candida* act as a physical scaffold, enabling the attachment and stabilization of bacterial species such as *Escherichia coli* and *Serratia marcescens*. This co-aggregation not only facilitates metabolic cross feeding but also creates a microenvironment that neutralizes the host’s innate immune response. The persistence of these reinforced biofilms in surgical specimens may also explain therapeutic refractoriness, as the dense matrix hinders the penetration of both endogenous antimicrobial peptides and exogenous pharmacological treatments (33).

An important factor that needs to be highlighted is the fact that the different responses to pharmacological treatment have also been associated with the intestinal mycobiome. Indeed, longitudinal data showed that successful response to anti-tumor necrosis factor (anti-TNF) or biologic therapy was associated with a partial restoration of the mycobiome; however, a “mycobiotic scar” (persistent low diversity and *Candida* dominance) remained in many patients, despite clinical remission. This imbalance may explain why many patients experience subclinical inflammation or early relapse, even when bacterial eubiosis appears to have been restored (17, 35, 56). Furthermore, non-responders to anti-TNF therapy exhibit a significantly more imbalanced fungal community at treatment baseline. Moreover, the mycobiome has also been associated with levels of inflammatory markers. In this sense, a positive correlation between fecal calprotectin levels (>250 μg/g) and *C. tropicalis* load has been reported (11, 35, 42, 43).

While this meta-analysis provides high-level evidence of fungal dysbiosis in IBD, several limitations must be addressed to contextualize its clinical translation. First, substantial methodological heterogeneity existed among the included studies, particularly regarding DNA extraction protocols, sequencing platforms, bioinformatic pipelines, and the use of ITS1 versus ITS2 fungal markers, which may have influenced taxonomic resolution and diversity estimates. Second, most studies were observational and cross-sectional in design, thus limiting causal inference between fungal dysbiosis and disease activity. From a technical perspective, the heterogeneity in high-throughput sequencing protocols – specifically, the lack of uniformity between ITS1 and ITS2 ribosomal DNA primers - prevents a direct comparison of absolute abundance metrics across different geographical populations. Third, important confounding factors - including diet, antibiotic exposure, proton pump inhibitor use, corticosteroid therapy, biologic treatment, and geographic variability - were inconsistently reported across cohorts. Additionally, the low biomass nature of fungal communities increases susceptibility to contamination and technical bias during sequencing analysis. Another important limitation is the difficulty in distinguishing transient dietary fungi from stable mucosa-associated colonizers, which may affect the interpretation of fecal mycobiome profiles. Finally, the relatively limited number of longitudinal and mechanistic studies currently available restricts the ability to establish whether fungal alterations represent drivers of inflammation or secondary consequences of intestinal disease. Standardized methodological frameworks and prospective multicenter studies are therefore urgently needed to improve reproducibility and facilitate clinical translation.

An additional limitation is that this systematic review was not prospectively registered in PROSPERO or another systematic review registry, and a formal protocol was not prepared or made publicly available before the review commenced. Prospective registration and protocol development improve methodological transparency by documenting the review objectives, eligibility criteria, outcomes, and planned analytical methods before study results are known, thereby facilitating the detection of potential deviations and reducing the risk of selective reporting or *post hoc* methodological decisions. Although the eligibility criteria, outcomes, data extraction procedures, risk-of-bias assessment, and synthesis methods reported in this manuscript were defined before study selection and were not subsequently modified, the absence of a prospectively registered and publicly accessible protocol prevents independent comparison between pre-specified and implemented methods. Therefore, the possibility of unrecognized methodological or selective reporting bias cannot be completely excluded. Future updates of this review should be prospectively registered and conducted according to a publicly accessible protocol, with any subsequent methodological amendments transparently documented. Nevertheless, several safeguards were implemented during the review process, including independent study screening and data extraction by two reviewers, resolution of disagreements by consensus with a senior reviewer, explicit eligibility criteria, and transparent reporting of the search and study-selection process. These measures do not replace prospective registration or a formal protocol, but they provide additional safeguards against errors and arbitrary decisions during study selection and data synthesis.

While the exclusion signature in CD appears to be robust, the more heterogeneous patterns observed in UC suggest that fungal dynamics in the colonic mucosa are more sensitive to localized inflammatory shifts than to systemic dysbiosis. These constraints highlight the urgent need for standardized mycobiome reporting guidelines to permit the transition of these fungal signatures from experimental observations to valid diagnostic tools in gastroenterology.

Beyond local mucosal confinement, the cross-kingdom disruption inherent to IBD triggers critical systemic consequences along the gut-liver axis (57, 58). Emerging high impact evidence indicates that intestinal barrier breakdown permits the translocation of microbial components and mycobiotic pathobionts, which directly orchestrate the polarization of resident lamina propria Th17 cells and the subsequent overproduction of IL-17 (59, 60). While IL-17 plays a dual role in maintaining mucosal barrier integrity under homeostatic conditions, its chronic, unconstrained production acts as a central driver of multi-organ inflammatory cascades (61). Specifically, recent mechanistic models have demonstrated that translocated gut-derived antigens travel via the portal circulation, activating hepatic Kupffer cells and stellate cells, thereby extending the Th17/IL-17 axis into the liver parenchyma (57, 61). This systemic immune dysregulation not only accentuates local epithelial destruction and continuous mucosal ulceration but also facilitates concomitant hepatic damage, providing a critical mechanistic link for extraintestinal manifestations and overlapping autoinflammatory biliary pathogenesis often co-occurring with refractory IBD phenotypes (24, 58).

Finally, the consolidation of the gut mycobiome as a cornerstone in the pathogenesis of IBD opens new horizons for clinical translation, moving from observational characterization to precision medicine. In the near future, the identification of specific fungal signatures or key pathobiont taxa, such as the expansion of *C. albicans* or *Malassezia* species alongside *Saccharomyces cerevisiae* depletion could serve as non-invasive biomarkers for disease stratification, prognostic evaluation, and the prediction of therapeutic responses, particularly in identifying non-responders to anti-TNF therapies prior to treatment initiation. However, translating these findings into routine gastroenterology requires overcoming significant technical bottlenecks, primarily the standardization of DNA extraction and the consensus on ITS1 versus ITS2 sequencing primers to eliminate baseline batch effects.

Looking forward, therapeutic strategies must evolve beyond the traditional bacterial-centric paradigm toward integrated, cross-kingdom interventions. This includes the development of targeted antifungal therapies against virulent hyphal morphotypes, the design of multi-kingdom probiotics capable of reversing the “mycobiotic scar”, and the disruption of polymicrobial biofilms to prevent therapeutic refractoriness and subclinical relapses. Prospective mechanistic studies in humanized models are urgently needed to fully decipher the causal networks of the gut–immune axis and to establish mycobiome modulation as a viable, safe, and standardized clinical reality.

## 4 Conclusions

The evidence synthesized in this review confirms that fungal dysbiosis is not a secondary consequence but a central driver of the inflammatory cascade in IBD, characterized by a loss of mycobiotic homeostasis that impairs the host’s immune tolerance and facilitates the exacerbation and perpetuation of the chronic inflammation. In this sense, the review suggests that the persistence of fungal dysbiosis beyond clinical remission represents a critical failure in current therapeutic strategies. This highlights that achieving full mucosal healing and long-term remission will require moving beyond a bacterial approach toward integrated, cross-kingdom precision medicine that places an emphasis on the mycobiome.

## Data Availability

The raw data supporting the conclusions of this article will be made available by the authors, without undue reservation.
